# Appropriate Contrast Enhancement Measures for Brain and Breast Cancer Images

**DOI:** 10.1155/2016/4710842

**Published:** 2016-03-31

**Authors:** Suneet Gupta, Rabins Porwal

**Affiliations:** ^1^Research Scholar, Mewar University, Chittorgarh 312901, Rajasthan, India; ^2^Department of Information Technology, Institute of Technology & Science (ITS), Ghaziabad 201007, Uttar Pradesh, India

## Abstract

Medical imaging systems often produce images that require enhancement, such as improving the image contrast as they are poor in contrast. Therefore, they must be enhanced before they are examined by medical professionals. This is necessary for proper diagnosis and subsequent treatment. We do have various enhancement algorithms which enhance the medical images to different extents. We also have various quantitative metrics or measures which evaluate the quality of an image. This paper suggests the most appropriate measures for two of the medical images, namely, brain cancer images and breast cancer images.

## 1. Introduction

We have different methods of enhancements which enhance the quality of an image [1, 2]. Enhancement can be done in such a way that the quality of image is upgraded visually or suiting to an application [3, 4]. Example of the latter case is medical image where the enhanced image must be more informative rather than being visually more pleasing. Therefore, to assess the quality of image, we have different measures of enhancement. There are some of the measures which suite images having different types of contents and attributes. Contrast based measures of enhancement methods, especially in spatial domain, use the intensities of pixels for calculation purpose. These spatial contrast measures are usually derived from Weber-Fechner law, Michelson contrast measure [5], or Contrast Ratio (CR). The measures PSNR, EME, AMEE [6], SOE, EC, and so forth are examples of such spatial contrast measures [7, 8]. These measures highly depend on image attributes like image content, background (uniform/nonuniform), lighting, texture, patterns which are periodic, single, or multiple targets, randomness, and also noise and distortions. Some measures or metrics are best suited to a set of attributes while other measures are best suited to other set of attributes. If a measure cannot properly tackle a particular image property, the metric or measure will not turn to prove a good measure for that image. Also, by looking at the image attributes, suitability of some measures can be suggested. In this paper, we will suggest the most appropriate or suitable measures of enhancement for brain and breast cancer images.

## 2. Basic Measures of Image Contrast

### 2.1. Michelson Contrast

This measure is generally used for images which have equivalent bright and dark features, for example, periodic patterns such as a sinusoidal grating. Michelson contrast is defined as

(1) $$\begin{array}{c} C_{M}=\frac{I_{\max}-I_{\min}}{I_{\max}+I_{\min}}, \end{array}$$

where *I*_max_ and *I*_min_ are the highest and lowest intensities.

### 2.2. Weber-Fechner Law

This measure is generally used for images with a large uniform background and a small test target. It is used for images whose average intensity is approximately equal to background intensity. It is given as

(2) $$\begin{array}{c} C_{W}=\frac{I_{t}-I_{b}}{I_{b}}, \end{array}$$

where*I*_*t*_ is the target intensity and *I*_*b*_ is the intensity of the immediate adjacent background.

### 2.3. Contrast Ratio

This measure is expressed in either linear or logarithmic form:

(3) CR=ItIb,log⁡CR=log⁡Itlog⁡Ib,

where *I*_*t*_ is the target intensity and *I*_*b*_ is the intensity of the immediate adjacent background.

### 2.4. Entropy

Entropy is calculated over entire image from the histogram of that image. It is a measure of randomness characterizing the texture of the image:

(4) $$\begin{array}{c} \text{Entropy}=-\sum p\times \ln\left(\;p\;\right), \end{array}$$

where*p* is the histogram count for a segment of image. Increasing the contrast in one portion of an image and decreasing it in another portion may result in similar entropy as the original image. This is because the entropy is calculated over the entire image.

## 3. Conventional Measures of Contrast

These measures are attractive because they are simple and easy to calculate. MSE and PSNR consider corresponding pair of pixels of two images for calculation purpose. The conventional measures actually calculate the difference between two images and therefore it can be used for both restoration and enhancement. These measures are taken from [5].

### 3.1. Mean Squared Error (MSE)

Let *A*(*m*, *n*) and *B*(*m*, *n*) be the original and enhanced image, respectively, where *m* × *n* represents the size of image. Then, MSE is defined as

(5) $$\begin{array}{c} \text{MSE}=\frac{1}{m\times n}\overset{m-1}{\underset{i=0}{\sum }}\;\overset{n-1}{\underset{j=0}{\sum }}{\left(\;B\left(\;i,j\;\right)-A\left(\;i,j\;\right)\;\right)}^{2}. \end{array}$$

### 3.2. Peak Signal-to-Noise Ratio (PSNR)

Let *A*(*m*, *n*) be an image with *L* gray levels and *m*×*n* being the size. Then, PSNR is defined as

(6) $$\begin{array}{c} \text{PSNR}=10\;\log_{10}\left(\;\frac{{\left(L-1\right)}^{2}}{\text{MSE}}\;\right). \end{array}$$

### 3.3. Absolute Mean Brightness Error (AMBE)

Let *A*(*m*, *n*) and *B*(*m*, *n*) be the original and enhanced image, respectively, where *m* × *n* represents the size of image. AMBE is calculated from the following equation:

(7) $$\begin{array}{c} \text{AMBE}=\left|\;E\left[\;Y\;\right]-E\left[\;X\;\right]\;\right|, \end{array}$$

where *E*[*Y*] and *E*[*X*] are mean gray levels of new and original image, respectively. They are given as

(8) EX=1m×n∑i=0m−1 ∑i=0n−1Ai,j,EY=1m×n∑i=0m−1 ∑i=0n−1Bi,j.

## 4. Complex Measures of Contrast

**Figure 1:**
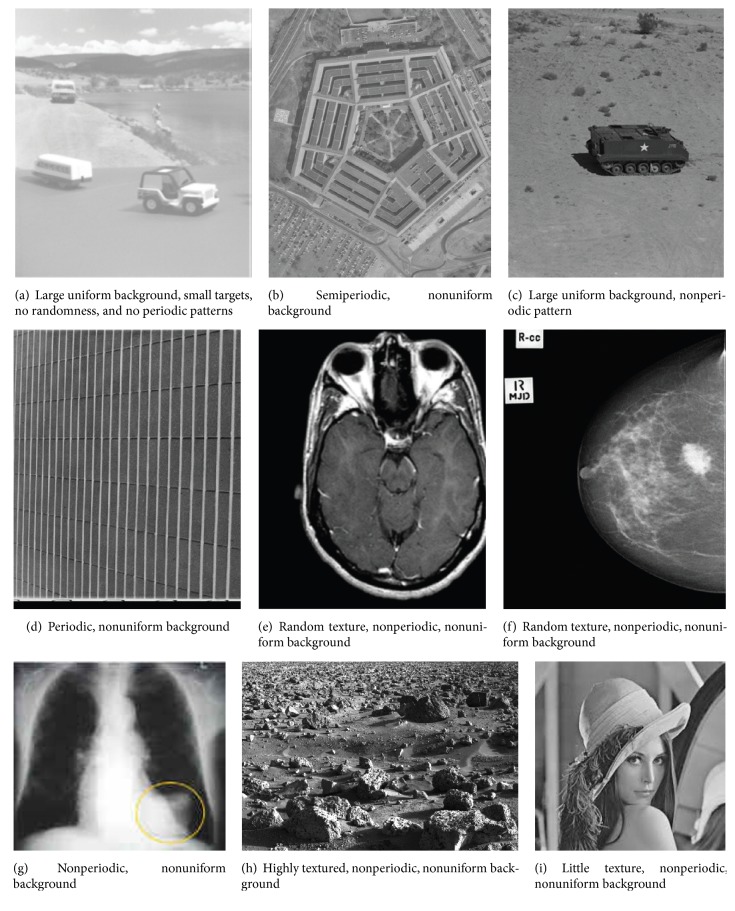
Images with different attributes.

These measures are based on the basic contrast measures and are highly sensitive to randomness, periodicity, uniform background, texture, target size, and noise. These measures are taken from [6]. These complex measures are always a good choice as good measures and, by looking at the attributes, one or two measures can be suggested. But doing it quantitatively is always a better option.  Figure 1 [6, 9–11] shows images having different attributes.

Let *A*(*m*, *n*) be an image which is split into *r* × *c* blocks, with *m*×*n* being the size of the image. Let each block be represented as *B*_*k*,*l*_(*i*, *j*), where *i* and *j* refer to *i*th row and *j*th column, respectively, inside each block. Let *I*_max;*k*,*l*_ and *I*_min;*k*,*l*_ be the maximum and minimum intensities in an image block *B*_*k*,*l*_(*i*, *j*).

### 4.1. Measure of Enhancement (EME)

The EME is defined as

(9) $$\begin{array}{c} {\text{EME}}_{rc}=\frac{1}{r\times c}\overset{r}{\underset{l=1}{\sum }}\;\overset{c}{\underset{k=1}{\sum }}\left[\;20\ln\left(\;\frac{I_{\max;k,l}}{I_{\min;k,l}}\;\right)\;\right]. \end{array}$$

As defined in (3), the CR of a block *B*_*k*,*l*_ is

(10) $$\begin{array}{c} {\text{CR}}_{k,l}=\frac{I_{\max;k,l}}{I_{\min;k,l}}. \end{array}$$

Therefore,

(11) $$\begin{array}{c} {\text{EME}}_{rc}=\frac{1}{r\times c}\overset{r}{\underset{l=1}{\sum }}\;\overset{c}{\underset{k=1}{\sum }}\left[\;20\ln\left(\;{\text{CR}}_{k,l}\;\right)\;\right]. \end{array}$$

The EME measure is suitable for images with attributes like noncomplex segments, uniform background in segments, small targets in segments, nonperiodic pattern in segments, and little to no randomness in segments.

### 4.2. Measure of Enhancement by Entropy (EMEE)

The EMEE is defined as

(12) EMEEαrc=1r×c∑l=1r ∑k=1cαImax⁡;k,lImin⁡;k,lαln⁡Imax⁡;k,lImin⁡;k,l.

As defined in (3), the CR of a block *B*_*k*,*l*_ is

(13) $$\begin{array}{c} {\text{CR}}_{k,l}=\frac{I_{\max;k,l}}{I_{\min;k,l}}. \end{array}$$

Therefore,

(14) EMEEαrc1r×c∑l=1r ∑k=1cαCRk,lαln⁡CRk,l=1r×c∑l=1r ∑k=1cEntropyCRα.

Therefore, EMEE measure is the entropy of Contrast Ratio for each block *B*_*k*,*l*_, scaled by *α* averaged over the entire image.

The EMEE measure is suitable for images with attributes like noncomplex segments or small size complex segments, nonperiodic patterns in segments, and randomness because of the inclusion of entropy. The more the value of *α*, the more the emphasis on entropy. The term *α* helps to handle more randomness.

### 4.3. Logarithmic Michelson Contrast Measure (AME)

The AME is defined as

(15) $$\begin{array}{c} {\text{AME}}_{rc}=\frac{-1}{r\times c}\overset{r}{\underset{l=1}{\sum }}\;\overset{c}{\underset{k=1}{\sum }}\left[\;20\ln\left(\;C_{M;k,l}\;\right)\;\right]. \end{array}$$

The AME measure is suitable for images with attributes like periodic patterns in segments and no randomness in texture. It cannot be used for images with uniform background and also images with segments of large uniform intensity.

### 4.4. Logarithmic AME by Entropy (AMEE)

The AMEE is defined as

(16) AMEErc=−1r×c∑l=1r ∑k=1cαImax⁡;k,l−Imin⁡;k,lImax⁡;k,l+Imin⁡;k,lα·ln⁡Imax⁡;k,l−Imin⁡;k,lImax⁡;k,l+Imin⁡;k,l.

Michelson contrast is given as

(17) $$\begin{array}{c} C_{M}=\frac{I_{\max}-I_{\min}}{I_{\max}+I_{\min}}. \end{array}$$

Therefore,

(18) AMEEαrc−1r×c∑l=1r ∑k=1cαCM;k,lαln⁡CM;k,l=1r×c∑l=1r ∑k=1cEntropyCM;k,lα.

Therefore, AMEE measure is the entropy of Michelson contrast for each block *B*_*k*,*l*_, scaled by *α* averaged over the entire image.

The AMEE measure is suitable for images with the attributes like surplus randomness in texture and periodic patterns in segments. It cannot handle images with large uniform background.

## 5. Other Measures of Contrast

Here, the first two measures are taken from [7] and the next two from [8].

### 5.1. Discrete Gray Level Energy (GLE)

The gray level energy measure basically refers to the way of distribution of gray levels. It is given as

(19) $$\begin{array}{c} \text{GLE}=\overset{k}{\underset{i=1}{\sum }}{p\left(i\right)}^{2}, \end{array}$$

where GLE represents the gray level energy with 256 bins and *p*(*i*) refers to the probability distribution functions of different gray levels, that is, the histogram count. In special case, GLE reaches its maximum value of 1 when the image has a constant value of gray level:

(20) $$\begin{array}{c} {\text{GLE}}_{\max}=\max\left\{\;\overset{k}{\underset{i=1}{\sum }}{p\left(i\right)}^{2}\;\right\}=1. \end{array}$$

### 5.2. Relative Entropy (RE)

Let *p* and *q* be two probability functions of processing images; then, RE of *p* with respect to *q* is defined as

(21) $$\begin{array}{c} \text{RE}=\overset{k}{\underset{i=1}{\sum }}p\left(\;i\;\right)\log_{2}\frac{p\left(i\right)}{q\left(i\right)}. \end{array}$$

Relative entropy is also called the Kullback Leibler distance.

### 5.3. Second-Order Entropy (SOE)

The first-order entropy is used as enhancement metric as it is less sensitive to noise and has greater response to gray level step. But it has the drawback of ignoring the correlation among pixels. Second-order derivatives enhance noise points much more and they are more sensitive to a line than a step and to a point than to a line.

The SOE as defined in [12] uses a cooccurrence matrix to consider the spatial correlation. Let *A* be an image of size *x* × *y* with *L* gray levels; then, element in the cooccurrence matrix *C* of size *L* × *L* which contains the information of correlation of the image is given as

(22) $$\begin{array}{c} c_{i,j}=\overset{y-1}{\underset{l=0}{\sum }}\;\overset{x-1}{\underset{k=0}{\sum }}\psi \left(\;l,k\;\right), \end{array}$$

where*c*_*i*,*j*_ is element at *i*th row and *j*th column of matrix *C* and

(23) ψl,k=1if  Al,k=i,  Al,k+1=jand/orAl,k=i,  Al+1,k=jψl,k=0otherwise.

The probability of cooccurrence *p*_*i*,*j*_ is computed as

(24) $$\begin{array}{c} p_{i,j}=\frac{c_{i,j}}{\sum_{k=0}^{L-1}\sum_{l=0}^{L-1}c_{i,j}} \end{array}$$

and the SOE is computed as

(25) $$\begin{array}{c} \text{SOE}=-\sum_{j}\sum_{i}p_{i,j}\;\log_{2}\left(\;p_{i,j}\;\right). \end{array}$$

### 5.4. Edge Content (EC)

Let *A*(*x*, *y*) be an image, where *x* is the row coordinate and *y* is the column coordinate. The gradient vector at any pixel position (*x*, *y*) is computed as

(26) $$\begin{array}{c} \delta A\left(\;x,y\;\right)=\left[\begin{array}{c} G_{x}\\ G_{y} \end{array}\right]=\left[\begin{array}{c} \frac{\partial }{\partial x}A\left(x,y\right)\\ \frac{\partial }{\partial y}A\left(x,y\right) \end{array}\right], \end{array}$$

where

(27) Gx=Ax,y−Ax+1,yGy=Ax,y−Ax,y+1.

Taking absolute value of image gradient as an indicator of contrast,

(28) $$\begin{array}{c} \left|\;\delta A\;\right|=\sqrt{G_{x}^{2}+G_{y}^{2}}. \end{array}$$

The measure EC is defined as

(29) $$\begin{array}{c} \text{EC}=\frac{1}{r\times c}\sum_{x}\sum_{y}\left|\;\delta A\left(\;x,y\;\right)\;\right|, \end{array}$$

where*r* × *c* is the size of image and

(30) 1≤x≤r,1≤y≤c.

Contrast changes exist over the entire image and EC takes into account all the contrast changes even for pixels which are very close (adjacent) to each other.

## 6. Enhancement Procedure 

To decide the most appropriate contrast measure, an enhancement procedure is required which enhances the image contrast to different levels. For this purpose, a Matlab function “*imadjust()*” [13] is used to increase the contrast to different levels. First, an image is read and if required converted to gray image. Let us call this image the original image. From the original image, an adjusted image of reasonably poor contrast is obtained. Let us call this image reference image. The reference image is created using the Matlab command as follows:

Reference Image, RI = imadjust(Original_Image, $\left[\begin{array}{cc} 0.2 & 0.8 \end{array}\right]$, $\left[\begin{array}{cc} 0.2 & 0.8 \end{array}\right]$);

where the second parameter, that is, the input range of gray levels, gets mapped to the third parameter, that is, the output range of gray levels. The full range of gray levels $\left[\begin{array}{cc} 0 & 1 \end{array}\right]$ for both input and output refers to $\left[\begin{array}{cc} 0 & 255 \end{array}\right]$.

Now, using the reference image, RI, different images of different levels of enhancement are obtained as follows:

*E*1 = imadjust(RI, $\left[\begin{array}{cc} 0.21 & 0.79 \end{array}\right]$, $\left[\begin{array}{cc} 0.19 & 0.81 \end{array}\right]$);
*E*2 = imadjust(RI, $\left[\begin{array}{cc} 0.22 & 0.78 \end{array}\right]$, $\left[\begin{array}{cc} 0.18 & 0.82 \end{array}\right]$);
*E*3 = imadjust(RI, $\left[\begin{array}{cc} 0.23 & 0.77 \end{array}\right]$, $\left[\begin{array}{cc} 0.17 & 0.83 \end{array}\right]$);
*E*4 = imadjust(RI, $\left[\begin{array}{cc} 0.24 & 0.76 \end{array}\right]$, $\left[\begin{array}{cc} 0.16 & 0.84 \end{array}\right]$);
*E*5 = imadjust(RI, $\left[\begin{array}{cc} 0.25 & 0.75 \end{array}\right]$, $\left[\begin{array}{cc} 0.15 & 0.85 \end{array}\right]$);

where *E*1, *E*2, *E*3, *E*4, and *E*5 are five enhanced versions of the reference image, RI. The enhancements of images are such that the contrast of RI < *E*1 < *E*2 < *E*3 < *E*4 < *E*5. It can be seen that for every next level of enhancement the input range has been narrowed down and the output range has been expanded resulting in contrast stretching and therefore more contrast.

## 7. Tests and Results

**Figure 2:**
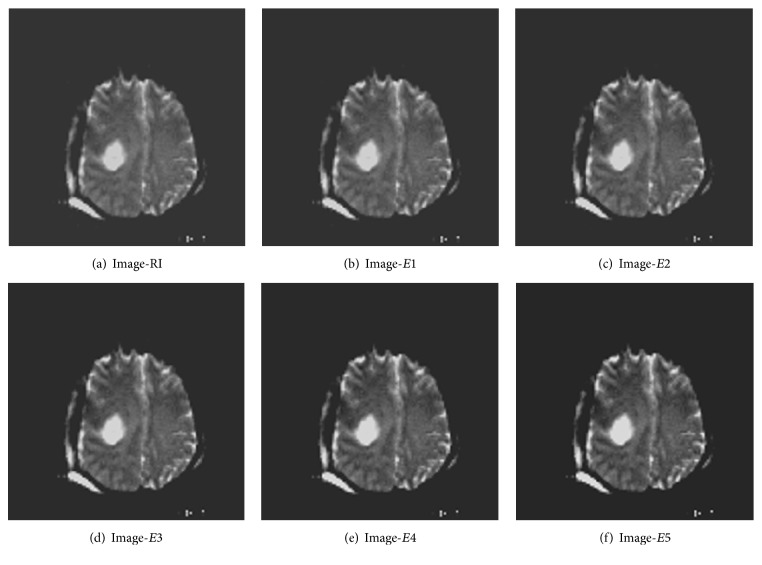
Different versions of one brain cancer image.

**Figure 3:**
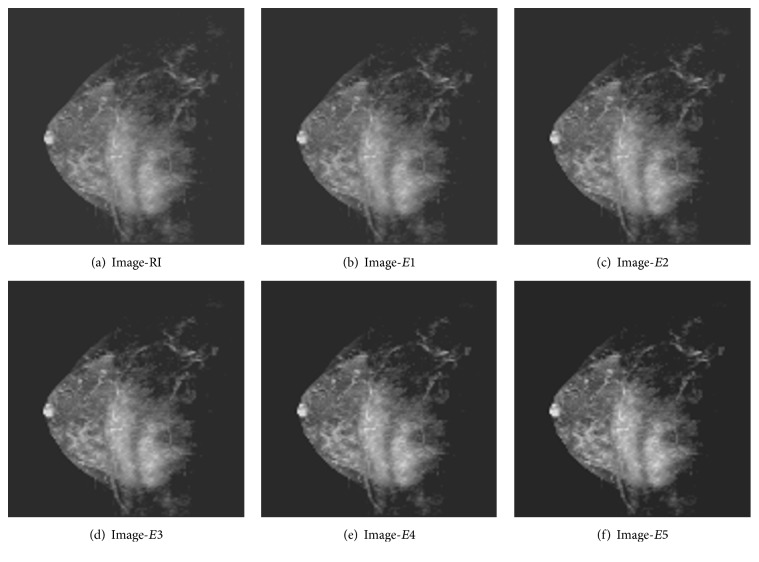
Different versions of one breast cancer image.

For the purpose of testing, twenty medical images are taken from [14], ten brain cancer images and ten breast cancer images. From each of these images, six versions, namely, one reference image (RI) and five enhanced images (*E*1 to *E*5), are created as discussed above. One example of each category, namely, brain cancer and breast cancer, is shown in Figures 2 and 3. Then, the average values of the contrast enhancement measures or metrics as mentioned above are computed and the results are shown in Tables 1 and 2. As far as the complex measures EME, EMEE, AME, and AMEE are concerned, the images are divided into blocks of size 16 × 16 and in case of EMEE and AMEE the value for *α* is taken as 0.1 as the randomness in the images of brain and breast cancer is quite low.

Defining contrast stretching (CS) quantitatively with respect to the reference image (RI) for the different enhanced versions of the images is as given in the following:

(31) $$\begin{array}{c} \text{CS}=\frac{\left(\left({\text{High}}_{\text{Out}}-{\text{Low}}_{\text{Out}}\right)/\left({\text{High}}_{\mathrm{In}}-{\text{Low}}_{\mathrm{In}}\right)\right)\text{ of Enhanced Image}}{\left(\left({\text{High}}_{\text{Out}}-{\text{Low}}_{\text{Out}}\right)/\left({\text{High}}_{\mathrm{In}}-{\text{Low}}_{\mathrm{In}}\right)\right)\text{ of Reference Image}}, \end{array}$$

where $\left[\begin{array}{cc} {\text{Low}}_{\text{Out}} & {\text{High}}_{\text{Out}} \end{array}\right]$ and $\left[\begin{array}{cc} {\text{Low}}_{\mathrm{In}} & {\text{High}}_{\mathrm{In}} \end{array}\right]$ are output range and input range of gray levels of the image. The denominator of (31) is 1 since

(32) HighOut−LowOutHighIn⁡−LowIn⁡  of Reference Image=0.8−0.20.8−0.2=1.0.

Therefore, (31) reduces to

(33) $$\begin{array}{c} \text{CS}=\frac{{\text{High}}_{\text{Out}}-{\text{Low}}_{\text{Out}}}{{\text{High}}_{\mathrm{In}}-{\text{Low}}_{\mathrm{In}}}\text{ of Enhanced Image}. \end{array}$$

Thus, CS with respect to RI for each of the enhanced versions is computed and given in Table 3.

Now, to find the most appropriate measure, the values of CS are taken as standard values and correlation between CS and each of the contrast metrics is computed and all are then compared. To calculate correlation, average values of the metrics as given in Tables 1 and 2 are taken and Pearson correlation [15] is used which is given as

(34) $$\begin{array}{c} r=\frac{n\left(\sum xy\right)-\left(\sum x\sum y\right)}{\sqrt{\left[n\sum x^{2}-{\left(\sum x\right)}^{2}\right]\left[n\sum y^{2}-{\left(\sum y\right)}^{2}\right]}}, \end{array}$$

where *x* and *y* are two variables between which the correlation, *r*, is to be found and *n* is the number of data. The two variables here would be CS and one enhancement measure. For illustration purpose, one calculation for MSE (brain cancer) using Table 4 has been shown.

Therefore,

(35) r=5×522.4878−6.1418×395.76685×7.612834−6.141825×51285.93−395.76682

or

(36) $$\begin{array}{c} r=0.9830. \end{array}$$

The value of *r* ranges from −1.0 to +1.0. When the value of *r* moves away from 0.0 to −1 or +1, the strength of association between the two variables increases; however, they are inversely associated if *r* is negative.  Table 5 shows the correlation values of the different contrast enhancement metrics used against brain and breast cancer images. As per Table 5, the most appropriate measure for brain cancer images is Edge Content (EC) and for breast cancer images EC stands as the second choice, with Absolute Mean Brightness Error (AMBE) being the first choice.

## 8. Conclusion with Discussion

From Table 5, we can see that all measures have quite high correlation values. The conventional measures MSE, PSNR, and AMBE are more used for restoration purpose and also they do not properly reflect the perceived visual quality of the image. Therefore, if we neglect the conventional measures, EC is the most appropriate measure for both brain and breast cancer images. Regarding complex measures EME, EMEE, AME, and AMEE, as they are sensitive to image attributes, they are a good choice for any type of image in general. To find the best among complex measures, we proceed with identifying the attributes of the image. Actually, there are different attributes of images which can be best handled by different measures; one measure cannot tackle all attributes equally well. Therefore, it is important to come to a conclusion of correct set of attributes the image in question has. Then, only a particular measure can be suggested. In this paper, both brain and breast cancer images have the following properties:

- Large uniform background.
- Random texture in the foreground but no randomness in the background.
- Nonperiodic pattern in the foreground but absence of any pattern in the background with the possibility to be considered periodic because the whole background is dark.
- Background containing noncomplex segments with the foreground containing complex segments.
- Background containing large area of uniform intensity which is absent in the foreground.

With these attributes, any recommendation would only be a compromise. Therefore, one way to make things simple is to examine only the foreground of the image as done in mammograms in [6]. Therefore, we forget the dark portion and consider the whole brain or breast as background and cells, tissues, lesions, and so forth as targets in the foreground. Now, the attributes of the image reduce to nonuniform background, random texture, and nonperiodic pattern. The best recommendation among the complex measures for these attributes is AMEE. AMEE cannot handle areas of uniform intensity well and we do have those types of areas but they are small, so we can go ahead with the recommendation.

The other aspect is when we consider the whole image, that is, when we consider the dark portion of the images as background and brain/breast as foreground, then irrespective of the attributes present in the image, the best among the complex measures can be picked up from Table 5 of this paper by seeing the correlation values. EME stands as the best choice whereas EMEE is a strong contender.

## Figures and Tables

**Table 1:** Average metrics values for brain cancer images.

	RI	*E*1	*E*2	*E*3	*E*4	*E*5
MSE	—	8.5517	26.5507	66.3417	109.7868	184.5359
PSNR	—	38.8112	33.8924	29.9151	27.7284	25.4730
AMBE	—	2.6961	4.7250	7.4828	9.5790	12.4371
EME	4.0391	4.3321	4.5948	4.9485	5.2719	5.6941
EMEE	0.0219	0.0236	0.0251	0.0272	0.0292	0.0317
AME	69.8246	68.6713	67.8564	66.5659	65.6326	64.2347
AMEE	0.2166	0.2138	0.2118	0.2087	0.2063	0.2029
GLE	0.5199	0.5226	0.5265	0.5289	0.5331	0.5363
RE	—	12.8020	12.9135	13.0003	13.1236	13.2628
SOE	3.7928	3.7594	3.7108	3.6806	3.6304	3.5931
EC	0.9306	0.9801	1.0253	1.0805	1.1305	1.1884

**Table 2:** Average metrics values for breast cancer images.

	RI	*E*1	*E*2	*E*3	*E*4	*E*5
MSE	—	9.0064	30.4972	73.9229	125.6293	207.1439
PSNR	—	38.5890	33.2949	29.4490	27.1487	24.9767
AMBE	—	2.4062	4.3634	6.8167	8.8055	11.3444
EME	6.0779	6.4918	6.8150	7.2779	7.6364	8.1497
EMEE	0.0325	0.0349	0.0368	0.0395	0.0417	0.0448
AME	54.3720	53.5601	53.2630	52.3984	52.0707	51.1420
AMEE	0.1791	0.1766	0.1753	0.1727	0.1714	0.1687
GLE	0.2531	0.2649	0.2825	0.2934	0.3113	0.3240
RE	—	8.9075	9.3810	9.6936	10.0909	10.4761
SOE	6.5488	6.4099	6.2002	6.0690	5.8614	5.7163
EC	1.6252	1.7027	1.7643	1.8450	1.9088	1.9889

**Table 3:** CS for different enhanced versions.

	*E*1	*E*2	*E*3	*E*4	*E*5
CS	1.0690	1.1429	1.2222	1.3077	1.4

**Table 4:** Illustration for calculation of correlation.

*x* (CS)	*y* (MSE)	*xy*	*x* ^2^	*y* ^2^
1.069	8.5517	9.14177	1.14276	73.1316
1.143	26.5507	30.3448	1.30622	704.940
1.222	66.342	81.0828	1.49377	4401.22
1.308	109.787	143.568	1.71008	12053.1
1.4	184.536	258.350	1.96	34053.5
6.142	395.767	522.4878	7.612834	51285.93

The last row of the table shows the summed up values of each column.

**Table 5:** Correlation values.

Metrics	Brain cancer images	Breast cancer images
MSE	0.9830	0.9846
PSNR	−0.9739	−0.9708
AMBE	0.9990	0.9993
EME	0.9992	0.9989
EMEE	0.9991	0.9989
AME	−0.9983	−0.9987
AMEE	−0.9986	−0.9947
GLE	0.9971	0.9960
RE	0.9987	0.9967
SOE	−0.9966	−0.9955
EC	0.9997	0.9991
